# Metallothionein III (MT3) is a putative tumor suppressor gene that is frequently inactivated in pediatric acute myeloid leukemia by promoter hypermethylation

**DOI:** 10.1186/1479-5876-12-182

**Published:** 2014-06-25

**Authors:** Yan-Fang Tao, Li-Xiao Xu, Jun Lu, Lan Cao, Zhi-Heng Li, Shao-Yan Hu, Na-Na Wang, Xiao-Juan Du, Li-Chao Sun, Wen-Li Zhao, Pei-Fang Xiao, Fang Fang, Yan-Hong Li, Gang Li, He Zhao, Yi-Ping Li, Yun-Yun Xu, Jian Ni, Jian Wang, Xing Feng, Jian Pan

**Affiliations:** 1 Department of Hematology and Oncology, Children’s Hospital of Soochow University, Suzhou, China; 2 Department of Gastroenterology, the 5th Hospital of Chinese PLA, Yin chuan, China; 3 Department of Cell and Molecular Biology, Cancer Institute (Hospital), Chinese Academy of Medical Sciences, Peking Union Medical College, Beijing, China; 4 Translational Research Center, Second Hospital, The Second Clinical School, Nanjing Medical University, Nanjing, China

**Keywords:** Metallothionein III, Pediatric acute myeloid leukemia, Methylation, Tumor suppressor

## Abstract

**Background:**

Acute myeloid leukemia (AML) is the second most common form of leukemia in children. Aberrant DNA methylation patterns are a characteristic feature in various tumors, including AML. Metallothionein III (MT3) is a tumor suppresser reported to show promoter hypermethylated in various cancers. However, the expression and molecular function of MT3 in pediatric AML is unclear.

**Methods:**

Eleven human leukemia cell lines and 41 pediatric AML samples and 20 NBM/ITP (Norma bone marrow/Idiopathic thrombocytopenic purpura) control samples were analyzed. Transcription levels of MT3 were evaluated by semi-quantitative and real-time PCR. MT3 methylation status was determined by methylation specific PCR (MSP) and bisulfite genomic sequencing (BSG). The molecular mechanism of MT3 was investigated by apoptosis assays and PCR array analysis.

**Results:**

The MT3 promoter was hypermethylated in leukemia cell lines. More CpG’s methylated of MT3 was observed 39.0% pediatric AML samples compared to 10.0% NBM controls. Transcription of MT3 was also significantly decreased in AML samples compared to NBM/ITP controls (*P* < 0.001); patients with methylated MT3 exhibited lower levels of MT3 expression compared to those with unmethylated MT3 (*P* = 0.049). After transfection with MT3 lentivirus, proliferation was significantly inhibited in AML cells in a dose-dependent manner (*P* < 0.05). Annexin V assay showed that apoptosis was significantly upregulated MT3-overexpressing AML cells compared to controls. Real-time PCR array analysis revealed 34 dysregulated genes that may be implicated in MT3 overexpression and apoptosis in AML, including *FOXO1*.

**Conclusion:**

MT3 may be a putative tumor suppressor gene in pediatric AML. Epigenetic inactivation of MT3 *via* promoter hypermethylation was observed in both AML cell lines and pediatric AML samples. Overexpression of MT3 may inhibit proliferation and induce apoptosis in AML cells. *FOXO1* was dysregulated in MT3-overexpressing cells, offering an insight into the mechanism of MT3-induced apoptosis. However, further research is required to determine the underlying molecular details.

## Introduction

Acute myeloid leukemia (AML) is a type of cancer in the myeloid cell. It is the most common form of acute leukemia in adults and the second most common form of leukemia in children after acute lymphoblastic leukemia (ALL) [1,2].The incidence of AML increases with age and is characterized by the rapid growth of abnormal white blood cells which accumulate in the bone marrow and interfere with the production of normal blood cells [3].

DNA methylation is an important regulator of gene transcription. It is an epigenetic modification that typically occurs at CpG (cytosine-phosphate-guanine) sites in mamalian cells. It is catalyzed by DNA methyltransferase which results in the conversion of the cytosine residue to 5-methylcytosine, leading to the formation of Me-CpG [4]. Alterations in DNA methylation frequently occur during cell development and are common in tumors. Aberrant DNA methylation patterns have been reported in a variety of cancers including AML [5,6]. Hypermethylation within the promoter regions of tumor suppressor genes leads to gene silencing, and global hypomethylation has also been recognized as a cause of oncogenesis [7,8]. As such, the role of methylation in carcinogenesis has been the focus of considerable research. However, the mechanisms leading to aberrant DNA methylation are poorly understood.

Genome-wide studies have been performed to obtain details on the genome and epigenome in various solid tumors. Examination of clonal evolution patterns have suggested that these may shape epigenetic dysregulation in AML. Investigations on genome-wide methylation in AML have shown variations in methylation patterns between AML and normal healthy tissues [9-11]. Although several studies have found that molecular subtypes in AML can exhibit highly distinct DNA methylation profiles [12,13], a small set of common genes have also been shown to exhibit consistent aberrant methylation across several hundred cases of AML [14,15], such as *PML-RARα* and *AML1-ETO*[12,13,16]. Together, these findings implicate distinct aberrant DNA methylation patterns in the pathogenetic processes in cancer, rather than evolving homogeneously across cancer types,

Deep sequencing of patients with different myeloid malignancies has revealed recurrent mutations in epigenetic regulator proteins, such as DNMT3A and TET2, and several major driver mutations in normal karyotype AML [17,18]. Functional studies of these mutations have revealed specific methylation signatures. These mutations carry prognostic relevance and are likely to be contributors to AML pathogenesis.

Metallothionein (MT) is a family of cysteine-rich, low molecular weight (500–14,000 Da) proteins [19]. Ten functional isoforms of MTs have been identified, which are divided into four classes (MT1–4) based on small differences in protein sequences and characteristics. MTs have been proposed to play important roles in protecting against DNA damage, apoptosis and oxidative stress. MT3 was first identified as a neuronal growth inhibitory factor [20]. *In vitro* studies have demonstrated that MT3 can inhibit neurite formation and survival in neurons [20]. It has been shown to protect against apoptotic neuronal death in the brains of patients with Alzheimer’s disease and in the hippocampus of SAMP8 mice, suggesting that MT3 may inhibit the development of neurodegeneration and may influence neuronal regeneration during the recovery process [21,22]. Its endogenous overexpression in glial and tumor cells stably transfected with MT3 was found to inhibit cell growth. In addition, MT3 plays a role in regulating lysosomal functions; in the absence of MT3, reductions in specific lysosomal enzymes associated with decreased autophagic flux have been reported [23].

In relation to cancer, downregulation of MT3 has been reported as one of 17 changes in gene expression which are associated with metastasis and poor clinical outcome in a range of solid tumors, including gastric cancer [24] and primary esophageal squamous cell carcinoma (SCC) [25]. This was supported by the observation that treatment with 5-aza-2'-deoxycytidine, an inhibitor of DNA methylation reduced the degree of methylation and increase the level of MT3 expression, in esophageal SCC cell lines [25]. Significant downregulation of MT3 has been most frequently reported in tumors that exhibiting MT3 methylation, suggesting that MT3 may act as a tumor suppresser via promoter hypermethylation [26].

However, reports on the methylation status of MT3 in the blood system are rare, and its expression and role in pediatric AML remains unclear. The aim of this study was to analyze the methylation profile and molecular function of MT3 in pediatric AML.

## Methods

### Cell lines

Leukemia cell lines HL-60, MV4-11, U937, DAMI and K562 were obtained from the American Type Culture Collection (ATCC). CCRF, Raji, Jurkat, 697 and SHI-1 cell lines (gifts from Professor Wang Jian-Rong, The Cyrus Tang Hematology center of Soochow University). All cell lines were maintained at 37°C in the RPMI 1640 (GibcoR, Life Technologies, Carlsbad, CA) supplemented with 10% fetal bovine serum (Invitrogen, Life Technologies, Carlsbad, CA).

### Patients and samples

Bone marrow specimens were obtained at the time of diagnosis during routine clinical assessment of 41 pediatric patients with AML, who presented at the Department of Hematology and Oncology, Children’s Hospital of Soochow University between 2000 and 2010. Ethical approval was provided by the Children’s Hospital of Soochow University Ethics Committee (No. SUEC2000-021), and informed consent was obtained from the parents or guardians. AML diagnosis was made in accordance with the revised French–American–British (FAB) classification. The main clinical and laboratory features of the patient cohort are summarized in Table 1. Additionally, bone marrow samples from 12 healthy donors and 8 patients with Idiopathic thrombocytopenic purpura (ITP) were analyzed as controls. Bone marrow mononuclear cells (BMNCs) were isolated using Ficoll solution within 2 h after bone marrow samples harvested and immediately subjected for the extraction of total RNA and genomic DNA.

**Table 1:** Correlation between MT3 methylation status and clinicopathological features in pediatric AML patients

**Clinical and pathologic features**	**Median (range)**			
	**Methylated (n = 16)**	**Unmethylated (n = 25)**	**Total patients (n = 41)**	***P*-value**
**Age** (years)	5.17 (1–13)	6.72 (1–11)	6.16 (1–13)	0.622
**Gender** (male/female ratio)	10/6	11/14	21/20	
**WBC:** median; range (10^9^/L)	14.1 (0.8–51.1)	18.2 (0.8–43.6)	16.6 (0.8–51.1)	0.757
**Hemoglobin:** median; range (g/L)	70.1 (32–176)	75.2 (32–107)	73.2 (32–176)	0.812
**Platelet count:** median; range (10^9^/L)	57.2 (12–310)	70.2 (23–273)	65.13 (12–310)	0.438
**FAB subtype (n = number of patients)**				
**M1**	2	4	6	
**M2**	5	5	11	
**M3**	7	4	11	
**M4**	3	2	5	
**M5**	3	5	8	0.733
**MT3 transcript**	10.65	19.23	16.37	0.049^*^

*FAB*, French-American-British; *WBC*, White blood cells. *P < 0.05.

### CD34 ^+^ cell purification

For CD34 ^+^cell selection, the Miltenyi immunoaffinity device (VarioMACS 130-046-703) was used according to the manufacturer’s instructions (Miltenyi Biotech, Auburn, CA). Briefly, the CD34^+^ cells are magnetically labeled with CD34 MicroBeads. Then, the cell suspension is loaded onto a MACSR Column which is placed in the magnetic field of a MACS Separator. The magnetically labeled CD34^+^ cells are retained within the column. The unlabeled cells run through; CD34^+^ cells were adsorbed on the magnetic poles. After removing the column from the magnetic field, the magnetically retained CD34^+^ cells can be eluted as the positively selected cell fraction.

### Sodium bisulfite modification of genomic DNA

High-molecular-weight genomic DNA was extracted from cell lines and biopsies by a conventional phenol/chloroform method. The sodium bisulphite modification procedure was as described [27] with slight modification. In brief, 600 ng of genomic DNA was denatured in 3 M NaOH for 15 min at 37°C, then mixed with 2 volumes of 2% low-melting-point agarose. Agarose/DNA mixtures were then pipetted into chilled mineral oil to form agarose beads. Aliquots of 200 μl of 5 M bisulphite solution (2.5 M sodium metabisulphite, 100 mM hydroquinone, both Sigma, USA) were added into each tube containing a single bead. The bisulphite reaction was then carried out by incubating the reaction mixture for 4 h at 50°C in the dark. Treatments were stopped by equilibration against 1 ml of TE buffer, followed by desulphonation in 500 μl of 0.2 M NaOH. Finally, the beads were washed with 1 ml of TE buffer and directly used for PCR.

### Methylation-specific PCR

The methylation status of the MT-3 promoter region was determined by methylation-specific PCR. Primers distinguishing unmethylated (U) and methylated (M) alleles were designed to amplify the sequence:

MT-3 M-forward: 5- TTAAGCGTATAAACGGAAAGAGC -3;

MT-3 M-reverse: 5- AAAACAAATCTCAAAATCCATATCG -3;

MT-3 U-forward: 5- TTTAAGTGTATAAATGGAAAGAGTGG -3;

MT-3 U-reverse: 5- AACAAATCTCAAAATCCATATCAAA -3.

Each PCR reaction contained 20 ng of sodium bisulphite-modified DNA, 250 pmol of each primer, 250 pmol deoxynucleoside triphosphate, 1 × PCR buffer, and one unit of ExTaq HS polymerase (Takara, Tokyo) in a final reaction volume of 20 μl. Cycling conditions were initial denaturation at 95°C for 3 min, 40 cycles of 94°C for 30 s, 60°C (M) or 58°C (U) for 30 s, and 72°C for 30 s. For each set of methylation-specific PCR reactions, in vitro-methylated genomic DNA treated with sodium bisulphite served as a positive methylation control. PCR products were separated on 4% agarose gels, stained with ethidium bromide and visualized under UV illumination. For cases with borderline results, PCR analyses were repeated.

### Bisulfite genomic sequencing

Bisulfite genomic sequencing (BGS) was performed as previously described [28]. BGS primers were from +427 to +672 including 15 CpGs. MT-3 F: 5- AGGGAGATTTGGTATTTTATTTTTT-3 and MT-3 R: 5- ACCTAACTATCTCTCCACATCCTAC-3. Amplified BGS products were TA-cloned; and five to six randomly chosen colonies were sequenced. DNA sequences were analyzed with QUMA Analyzer. (http://quma.cdb.riken.jp/).

### Leukemia cell cells treated with 5-aza-2'-deoxycytidine

De-methylation was induced with 5-aza-dC (5-Aza, Sigma-Aldrich, St Louis, MO, USA) treatment at a concentration that induced de-methylation of the DNA without killing the cells. Culture media for HL-60 and MV4-11 cells contained 5 μM 5-Aza. DNA and RNA were extracted after 72 hours of 5-Aza treatment for the following analysis.

### Quantitative reverse-transcription PCR for MT-3

Quantitative real-time PCR was performed to determine the expression levels of MT-3 genes. Total RNA was reverse transcribed using the Reverse Transcription Kit, according to the manufacturer’s protocol (Applied Biosystems Inc., Foster City, CA). The real time PCR primers used to quantify GAPDH expression were: F: 5′-AGAAGGCTGGGGCTCATTTG-3′ and R: 5′-AGGGGCCATCCACAGTCTTC-3′ and for MT-3 were: F: 5′-ACACACAGTCCTTGGCACAC-3′ and R: 5′-AAGTGCGAGGGATGCAAAT-3′. Expression of MT-3 was normalized to endogenous GAPDH expression.

### MT-3 lentiviral expression constructs and lentivirus production

Briefly, an approximately 250 bp fragment containing the human MT-3 gene was directly synthesized, and cloned into the pMD18-T vector. Positive clones were confirmed by sequencing and subcloned into the pLVX-IRES-ZsGreen vector (Clontech). The vector plasmids, pLVX-IRES-ZsGreen1, pLP1, pLP2 and pLP/VSVG were amplified in E.Coli and purified using the Endofree Maxiprep Kit (Qiagen). 270 μg of transfer vector, 176 μg of pLP1, 95 μg of pLP/VSVG and 68 μg of pLP2 was mixed with 0.25 M CaCl2 (Sigma) and added to same volume of 2 × HEPES (Sigma) and mixed while bubbling for 20 min to allow a precipitate to form. This was then added to a 175 cm2 flask of approximately 60% confluent 293 T cells containing 20 mL DMEM supplemented with 10% fetal calf serum, 100 U/mL penicillin, 100 μg/mL streptomycin and 2 mM glutamine and incubated for 48 h at 37°C in 5% CO2. The supernatant was centrifuged at 1,700 g for 10 min to pellet cell debris, and ultracentrifuged at 121,603 g for 2 h. The pellet containing concentrated virus was resuspended in DMEM without supplements and stored at -80°C.

### Cell proliferation analysis

Acute myeloid leukemia cells were seeded in 96-well plates at 2 × 10^4^ cells per well. 20 ul CCK-8(Dojindo Molecular Technologies, Japan) was added to each well and incubated at 37°C for a further 4 hours. The optical density (OD) values were measured at 450 nm on a scanning multi-well spectrophotometer (BioRad Model 550, USA). Compared with the control group, Cell proliferation was calculated as proliferation values. All experiments were performed in triplicate and repeated twice. The results were analyzed using ANOVA and the Student-Newman-Keuls tests, *p* < 0.05 were considered significant.

### Apoptosis assay

Apoptosis assay was according to the manual operation of BD Annexin V Staining Kit (Cat: 556420, BD Biosciences, Franklin Lakes and NJ USA). Briefly, wash cells twice with cold PBS and then resuspend cells in 1 × Binding Buffer at a concentration of ~1 × 10^6^ cells/ml. Transfer 100 μl of the solution (~1 × 10^5^ cells) to a 5 ml culture tube. Add Annexin V and PI 5 μl/test. Gently mix the cells and incubate for 15 min at RT in the dark. Add 400 μl of 1 × Binding Buffer to each tube. Analyze by flow cytometry as soon as possible (within 1 hour).

### Western blot analysis

For western blot analysis, cellular proteins were extracted in 40 mM Tris–HCl (pH 7.4) containing 150 mM NaCl and 1% (v/v) Triton X-100, supplemented with a cocktail of protease inhibitors. Equal amounts of protein were resolved on 12% SDS-PAGE gels, and then transferred to a PVDF membrane (Millipore, Bedford, MA). Blots were blocked and then probed with antibodies against MT-3(1:1000, Abcam, Cambridge, MA Office, USA), PARP (1:1000, Cell Signaling Technology, Inc. Danvers, MA), FOXO1 (1:1000, Cell Signaling Technology, Inc. Danvers, MA), CDKN1A (1:1000, Cell Signaling Technology, Inc. Danvers, MA), GAPDH (1:5000, Sigma, St. Louis, MO). After washing, the blots were incubated with horseradish peroxidase-conjugated secondary antibodies and visualized by enhanced chemiluminescence kit (Pierce, Rockford, IL). Protein bands were visualized after exposure of the membrane to Kodak X-ray film.

### Real-time PCR array analysis

For RNA extraction, cells were immediately submerged in 2 ml Trizol (Invitrogen Co., NY, USA), stored at -80°C until further processed. A volume of 1 ml of each sample was spun at 4°C for 15 min at 12,000 g to remove debris and DNA, 1 ml of supernatant was mixed with 200 ul chloroform, shaken for 15 seconds, incubated at Room Temperature for 2–3 minutes and spun for 10 minutes at 12,000 g at 4°C. RNA was precipitated by adding 500 ul of the aqueous phase to an equal volume of isopropanol and spun at 14,000 g at 4°C for 10 minutes. RNA was washed with 75% ethanol, spun at 14,000 g at 4°C for 10 minutes, dried and resuspended in 40 ul DEPC-treated H2O. The final RNA concentration was determined using a spectrophotometer (Nanodrop 2000) and the purity was assessed by agarose gel electrophoresis. cDNA synthesis was performed on 4 ug of RNA in a 10 ul sample volume using SuperScript II reverse transcriptase (Invitrogen Co., NY, USA) as recommended by the manufacturer. The RNA was incubated with 0.5 ug of oligo (dT)12–18mers primers (Invitrogen Co., NY, USA) for 7 minutes at 70°C and then transferred onto ice. Then, 9 ul of a master mix containing 4 ul of SuperScript II buffer, 2 ul of 0.1 M DTT, and 1 ul each of dNTPs stock (10 mM), Rnasin (40 UI) and SuperScript II were added to the RNA sample, spun and incubated at 42°C for 60 min followed by 5 min at 70°C to inactivate the enzyme. cDNA was stored at -20°C. Real-time PCR array (SABioscience Human Apoptosis PCR Array PAHS-3012) analysis was performed in a total volume of 20 ul including 2ul of cDNA, primers (0.2 mM each) and 10 ul of SYBR Green mix (Roche Co., Basel, Switzerland). Reactions were run on an Light cycler 480 using the universal thermal cycling parameters (95°C 5 min, 45 cycles of 10 sec at 95°C, 20 sec at 60°C and 15 sec at 72°C; melting curve: 10 sec at 95°C, 60 sec at 60°C and continues melting). Results were obtained using the sequence detection software Light cycler 480 and analyzed using Microsoft Excel. For all samples melting curves were acquired for quality control purposes. For gene expression quantification, we used the comparative Ct method. First, gene expression levels for each sample were normalized to the expression level of the housekeeping gene encoding Glyceraldehydes 3-phosphate dehydrogenase (GAPDH) within a given sample (-⊿Ct); the relative expression of each gene was calculated with 10^5^ *Log2(-⊿Ct). The difference between the MT-3 over-expression samples compared to the control samples was used to determine the 10^6^ *Log2(-⊿Ct). Statistical significance of the gene expression difference between the MT-3 over-expression and the control samples was calculated with the T-test using SPSS 11.5 software.

#### Statistical analysis

SPSS v11.5 (SPSS Inc., Chicago, IL) was used for statistical analysis. Data are presented as means ± standard deviation. Group t-test was used to compare the expression of MT-3 between DMSO group and 5-Aza group. Statistical significance between methylated sample data and clinical pathological features of AML patients were analyzed by Pearson chi-square test or Fisher’s exact test. Statistical significance of MT-3 expression among NBM and pediatric AML groups was determined using one-way ANOVA. A p < 0.05 was considered statistically significant.

## Results

### The MT3 promoter is hypermethylated in AML cells

**Figure 1:**
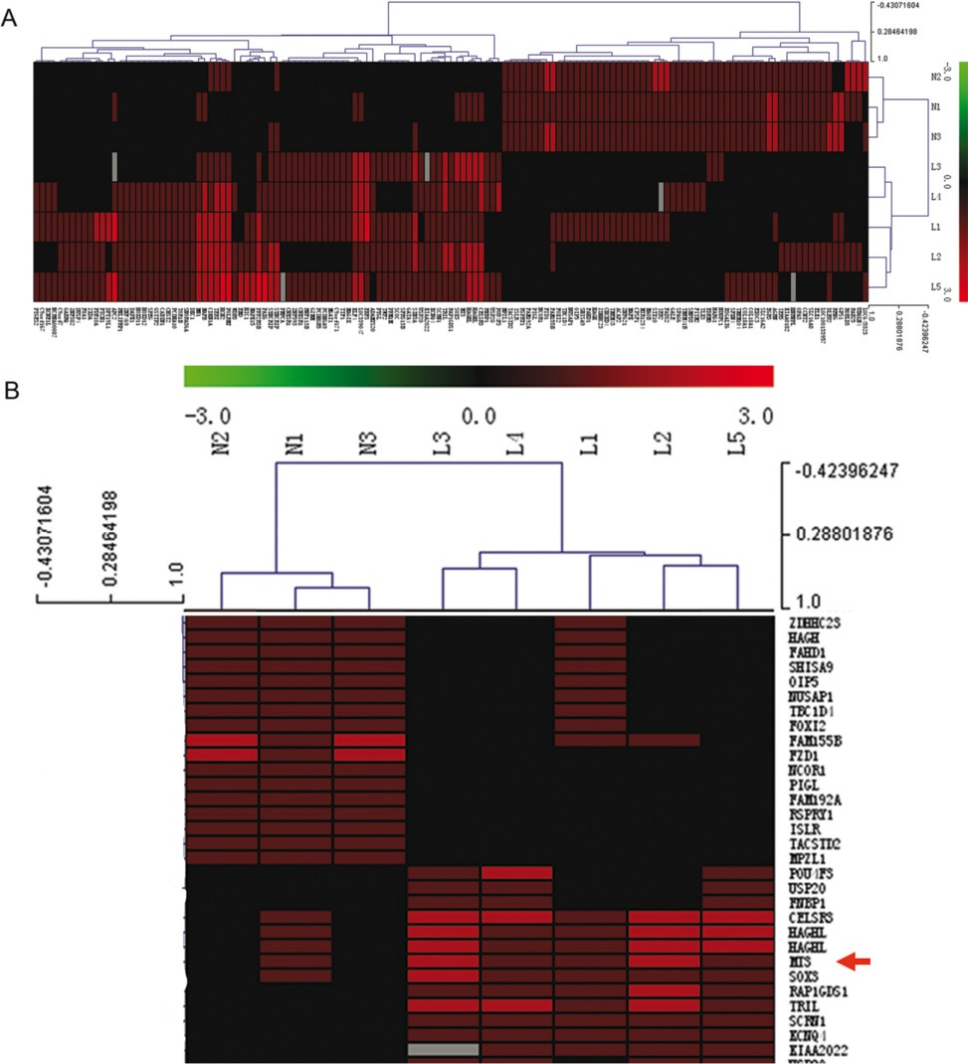
**Analysis of promoter methylation in pediatric AML by NimbleGen Human DNA Methylation arrays.** Analysis of the methylation status of genes in five pediatric AML samples (L1, L2, L3, L4 and L5) and three NBM samples (N1, N2, and N3) using NimbleGen Human DNA Methylation arrays shows that the MT3 promoter is significantly methylated in AML samples (5/5) and unmethylated in NBM samples (1/3). **(A)** Each red box represents the number of methylation peaks (PeakScore) overlapping the promoter region for the corresponding gene. The PeakScore is defined as the average -log10 (*P*-value) from probes within the peak. **(B)** The scores reflect the probability of positive methylation enrichment.

**Figure 2:**
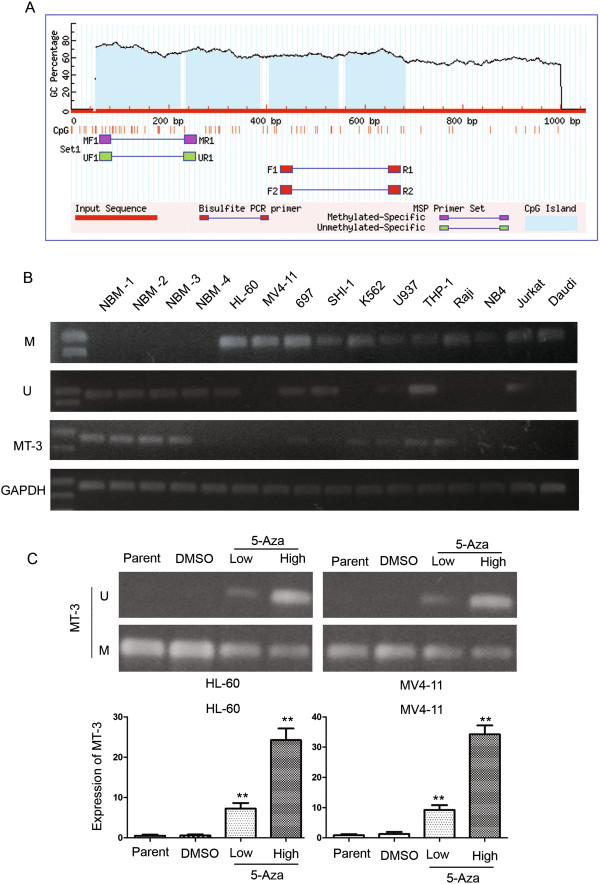
**The MT3 promoter is methylated in AML cell lines. (A)** Four CpG island regions can be identified in the promoter of MT3. **(B)** MSP analysis of the methylation status of MT3 in leukemia cell lines shows that the promoter is hypermethylated in 7/11 cell lines. M and U represent MSP results using primer sets for methylated and unmethylated MT3 genes, respectively. **(C)** PCR analysis showed that methylation status of MT3 was decreased in leukemia cells following 5-Aza treatment compared to control cells treated with DMSO. The transcript level of MT3 is significantly upregulated in HL-60 cells and MV4-11cells treated with 5-Aza compared to those treated with DMSO. *P < 0.05; **P < 0.01.

Our long-term research is focused on epigenic modification in pediatric AML and we have found a series of abnormal methylated genes related with AML [29,30]. In this study we conducted CpG island array analysis to explore promoter methylation in pediatric AML. The hypermethylated and hypomethylated genes between AML and NBM were clustered in Figure 1 and Additional file 1. The results implied that the MT3 promoter was hypermethylated in AML. Subsequent analyses identified four CpG islands in the MT3 promoter region (Figure 2A). Therefore, we conducted an MSP assay in 11 leukemia cell lines using a primer for MSP analysis that encompassed the CpG islands in the MT3 promoter. Our results showed that the MT3 promoter was hypermethylated in 7/11 leukemia cell lines, with the highest methylation levels observed in HL-60, MV4-11 SHI-1, U937 and K562 cells; whereas it was unmethylated in 4/11 cell lines, 697, SHI-1, THP-1 and Jurkat (Figure 2B). To confirm methylation of the MT3 promoter, we treated the leukemia cell lines with 5-Aza. This demethylation reagent is an epigenetic modifier that inhibits DNA methyltransferase activity resulting in hypomethylation and gene activation. PCR analysis showed that methylation status of MT3 was decreased in leukemia cells following 5-Aza treatment compared to control cells treated with DMSO. Our results showed that MT3 expression was significantly upregulated in leukemia cells following 5-Aza treatment compared to control cells treated with DMSO (Figure 2C): MT3 expression was upregulated 39.8 fold in HL-60 cells (24.27 vs. 0.61, respectively; *P* = 0.013); and 26.8 fold in MV4-11 cells (34.27 vs. 1.28, respectively; *P* = 0.006).

### The MT3 promoter was methylated in patients with pediatric AML

**Figure 3:**
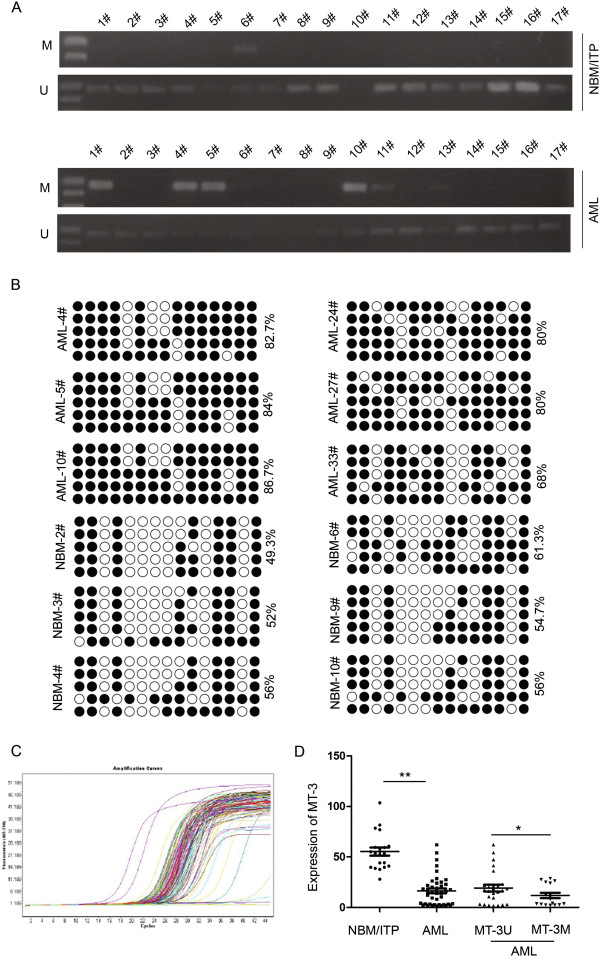
**MT3 is inactivated by promoter hypermethylation in AML cell lines. (A)** MSP analysis of the methylation status of MT3 shows aberrant methylation in pediatric AML samples compared to NBM/ITP control samples. Aberrant methylation of MT3 was observed in 39.0% (16/41) of the pediatric AML samples compared to 10.0% (2/20) of the NBM control samples. M and U represent MSP results using primer sets for methylated and unmethylated MT3 genes, respectively. **(B)** BGS results of three AML samples and three NBM samples show that CpG islands are methylated in the AML samples and unmethylated in the NBM control samples. **(C)** Real-time PCR analysis of the transcript levels of MT3 in 41 pediatric AML samples and 20 NBM control samples. **(D)** Quantification shows that MT3 expression is significantly decreased in the AML samples compared to the NBM/ITP control samples. Furthermore, AML patients with methylated MT3 (n = 16) show lower MT3 transcript levels than those unmethylated MT3 (n = 25).

In order to examine the methylation status in the MT3 promoter in pediatric AML, we obtained samples from 41 patients with pediatric AML and 20 control patients with NBM/ITP. Aberrant MT3 methylation was observed in 39.0% (16/41) of the pediatric AML samples compared to 10.0% (2/20) of the NBM control samp (Figure 3A). Six NBM samples and six AML samples were selected for further analysis by BGS (Figure 3B). Consistent with the MSP results, these confirmed that the CpG islands in the MT3 promoter were methylated in the AML samples (68%-86.7%); whereas they were unmethylated in the NBM samples (49.3%-61.3%).

Examination of the clinicopathologic characteristics in patients with pediatric AML revealed that there were no significant differences in clinical features, such as sex, age, initial hemoglobin level, white blood cell counts, platelet counts and chromosomal abnormalities between those with methylated MT3 and those with unmethylated MT3 (Table 1).

Real-time qPCR was employed to examine the transcript levels of *MT3* in the 41 pediatric AML samples and 20 NBM/ITP control samples (Figure 3C; Table 1). MT3 expression was found to be significantly decreased in the AML samples compared to the control samples (16.37 ± 15.09 vs. 55.25 ± 18.34; *P* < 0.001). Further analysis of the AML samples showed that 16/41 patients with pediatric AML displayed methylated MT3 compared to 25/41 patients with unmethylated MT3 (Table 1). Furthermore, those with methylated MT3 showed significantly lower levels of MT3 expression compared to those with unmethylated MT3 (10.65 ± 10.19 vs. 19.23 ± 16.93; *P* = 0.049; Figure 3D). In summary the hypermethylation status of the MT3 promoter in samples from patients with pediatric AML was consistent with results in human myeloid leukemia cell lines.

### Overexpression of MT3 inhibited proliferation and induced apoptosis in leukemia cells

**Figure 4:**
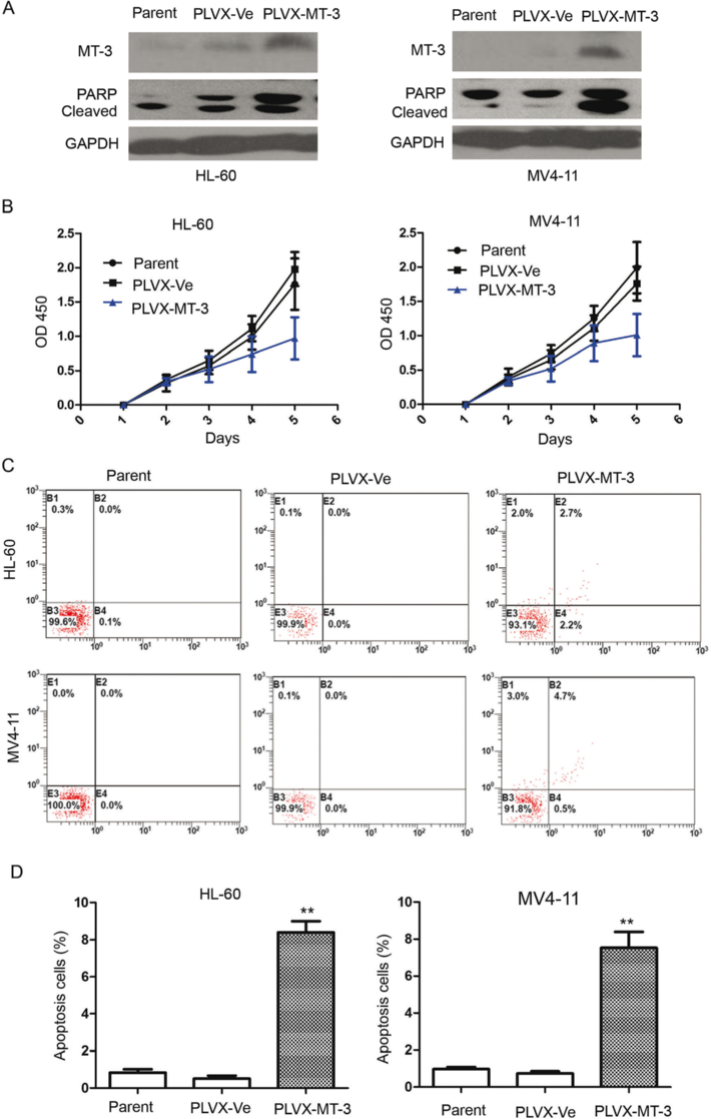
**Overexpression of MT3 inhibited proliferation an induced apoptosis in leukemia cells. (A)** Transfection with MT3 lentivirus PLVX-MT3 significantly upregulates expression of MT3 in AML cells compared to mock transfected cells. **(B)** CCK-8 assays show that transfection with MT3 lentivirus inhibits proliferation in HL-60 and MV4-11 cells in a dose-dependent manner compared to mock transfected cells. **(C)** The number of cells displaying apoptotic features is higher in the HL-60 an MV4-11 cells transfected with PLVX-MT3compared to the mock transfected cells. **(D)** Quantification shows that the percentage of apoptotic cells in PLVX-MT3 transfected group is significantly higher compared to the percentage in the mock control group. *P < 0.05; **P < 0.01.

Transfection of the PLVX-MT3 lentivirus into HL-60 and MV4-11 leukemia cells was found to significantly upregulate expression of MT3 (Figure 4A) and significantly inhibit cell proliferation (Figure 4B). A CCK-8 assay in HL-60 and MV4-11cells showed that the inhibition rate at 5 days post-transfection was 43.7 ± 30.1% and 51.1 ± 26.8% in MT3-overexpessing cells compared to the mock transfection group (*P* < 0.05).

To determine whether MT3 induced apoptosis in leukemia cells, we performed an Annexin V assay in HL-60 and MV4-11 leukemia cells following transfection (Figure 4C and D). The results showed that the proportion of apoptotic cells in the MT3-overexpressing cells (PLVX-MT3) was significantly greater than that in the control group (PLVX-Ve): HL-60 (8.40% ± 1.04% vs. 0.5% ± 0.3%, respectively; *P* = 0.003); and MV4-11 (7.53% ± 1.49% vs. 0.73 ± 0.21%, respectively; *P* = 0.014).To further investigate the apoptotic effect of MT3 in HL-60 and MV4-11 cells, we investigated the expression levels of cleaved PARP, a marker of apoptosis, by Western blotting. The results were consistent with the Annexin V data, confirming that MT3 induced apoptosis in leukemia cells (Figure 4A).

### Dysregulation of apoptosis-related genes in HL-60 cells overexpressing MT3

**Figure 5:**
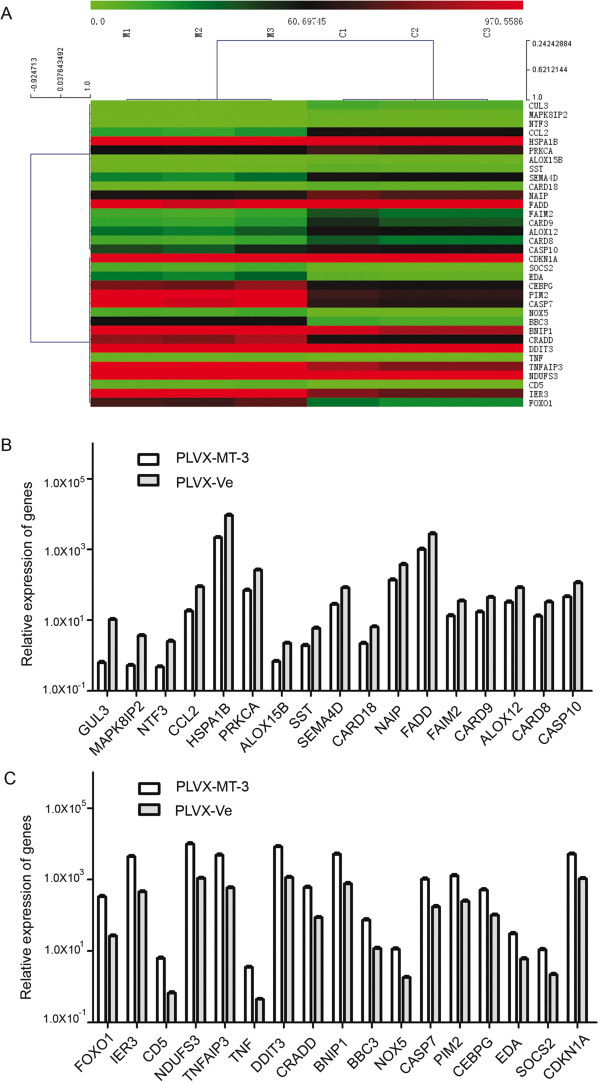
**Real-time PCR array analysis showing dysregulated genes implicated in MT3 overexpression. (A)** In order to identify genes associated with MT3 overexpression and apoptosis in AML cells, we analyzed and clustered the expressions of 370 key genes involved in apoptosis using the SABioscience Human Apoptosis PCR Array PAHS-3012 kit. **(B)** Relative expression of the genes most downregulated in MT3-overexpressing AML cells compared to mock transfected cells. **(C)** Relative expression of the genes most upregulated in MT3-overexpressing cells compared to mock transfected cells.

**Figure 6:**
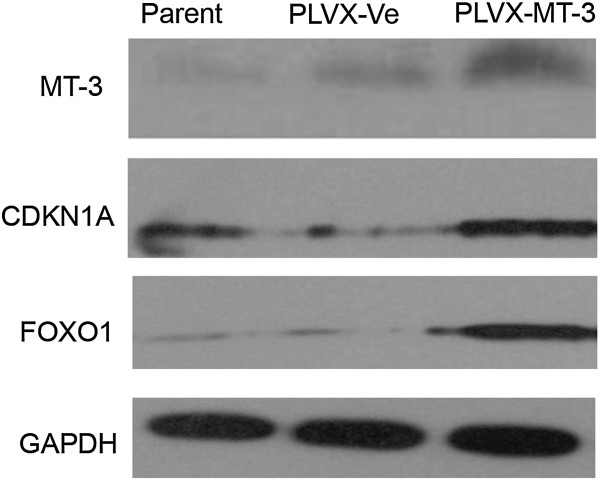
**Western-blot analysis showing up-regulation of FOXO1 and CDKN1A implicated in MT3-overexpressing.** Western-blot analysis showed that expression of CDKN1A and FOXO1 is significantly up-regulated in MT3-overexpressing cells compared to mock transfected cells.

In order to identify the apoptosis-related genes implicated in MT3 overexpression in HL-60 cells, we analyzed expression and clustering of 370 key genes associated with apoptosis by real-time PCR array analysis, cells harboring empty vector or a vector overexpressing MT3 were subjected to real-time PCR array (Figure 5A). The genes most significantly downregulated or upregulated are shown in Figures 5B and 5C, respectively. Examination of the array data revealed that 17 genes were significantly upregulated and 17 genes were significantly downregulated in the MT3-overexpressing group compared to the control group (Tables 2 and 3, respectively). These included *TNF* (tumor necrosis factor) ligands and their receptors, members of the bcl-2 family, BIRC (baculoviral IAP repeat) domain proteins, CARD (caspase recruitment domain) proteins, death domain proteins, TRAF (TNF receptor-associated factor) domain proteins, and caspases. *FOXO1* was found to be the most upregulated gene in MT3-overexpressing group. The up-regulation of FOXO1 and CDKN1A in MT3-overexpressing group was vivificated with western-blot analysis (Figure 6).

**Table 2:** Genes up regulated in HL-60 cells treated with PLVX-MT-3 compared with control group

	**Gene**	**Description**	**Control**	**MT-3**	**FC**	**P**
1	FOXO1	forkhead box O1	26.4281	331.0229	12.5254	0.010474
2	IER3	immediate early response 3	452.3756	4374.4713	9.6700	0.010816
3	CD5	CD5 molecule	0.6702	6.2407	9.3121	0.010872
4	NDUFS3	NADH dehydrogenase (ubiquinone) Fe-S protein 3	1081.0850	9828.4023	9.0912	0.010909
5	TNFAIP3	tumor necrosis factor, alpha-induced protein 3	587.6202	4806.8271	8.1802	0.011077
6	TNF	tumor necrosis factor	0.4428	3.4995	7.9040	0.011134
7	DDIT3	DNA-damage-inducible transcript 3	1145.0392	8216.9381	7.1761	0.011304
8	CRADD	CASP2 and RIPK1 domain containing adaptor with death domain	86.0764	598.6941	6.9554	0.011361
9	BNIP1	BCL2/adenovirus E1B 19 kDa interacting protein 1	763.0465	5084.0947	6.6629	0.011443
10	BBC3	BCL2 binding component 3	11.7979	73.7195	6.2486	0.01157
11	NOX5	NADPH oxidase, EF-hand calcium binding domain 5	1.8139	11.2984	6.2286	0.011576
12	CASP7	caspase 7, apoptosis-related cysteine peptidase	172.3255	1016.9670	5.9014	0.011688
13	PIM2	pim-2 oncogene	247.2885	1270.8713	5.1392	0.012
14	CEBPG	CCAAT/enhancer binding protein (C/EBP), gamma	100.8660	510.3751	5.0599	0.012038
15	EDA	ectodysplasin A	6.0342	30.4256	5.0422	0.012047
16	SOCS2	suppressor of cytokine signaling 2	2.1896	10.8844	4.9710	0.012082
17	CDKN1A	cyclin-dependent kinase inhibitor 1A	1057.6600	5124.4420	4.8451	0.012147

**Table 3:** Genes down regulated in HL-60 cells treated with PLVX-MT-3 compared with control group

	**Gene**	**Description**	**Control**	**MT-3**	**FC**	**P**
1	CUL3	cullin 3	10.4408	0.6273	0.0601	0.008493
2	MAPK8IP2	mitogen-activated protein kinase 8 interacting protein 2	3.6692	0.5249	0.1431	0.009149
3	NTF3	neurotrophin 3	2.5110	0.4725	0.1882	0.009344
4	CCL2	chemokine (C-C motif) ligand 2	88.1973	18.1459	0.2057	0.009402
5	HSPA1B	heat shock 70 kDa protein 1B	9152.4687	2147.2853	0.2346	0.00949
6	PRKCA	protein kinase C, alpha	259.7601	68.8275	0.2650	0.009593
7	ALOX15B	arachidonate 15-lipoxygenase, type B	2.2320	0.6779	0.3037	0.009776
8	SST	somatostatin	5.9015	1.9173	0.3249	0.009921
9	SEMA4D	sema domain, immunoglobulin domain 4D	82.5241	27.6746	0.3354	0.010009
10	CARD18	caspase recruitment domain family, member 18	6.4031	2.1791	0.3403	0.010055
11	NAIP	NLR family, apoptosis inhibitory protein	374.1656	136.0874	0.3637	0.010316
12	FADD	Fas (TNFRSF6)-associated via death domain	2746.1986	1001.6552	0.3647	0.010329
13	FAIM2	Fas apoptotic inhibitory molecule 2	34.8320	13.2906	0.3816	0.010573
14	CARD9	caspase recruitment domain family, member 9	44.1602	16.8715	0.3821	0.01058
15	ALOX12	arachidonate 12-lipoxygenase	82.6640	32.4829	0.3930	0.010768
16	CARD8	caspase recruitment domain family, member 8	32.5951	12.9265	0.3966	0.010835
17	CASP10	caspase 10, apoptosis-related cysteine peptidase	114.4506	45.4485	0.3971	0.010845

## Discussion

In this study, we found that the MT3 promoter was hypermethylated in pediatric AML. Our results showed that the MT3 promoter was hypermethylated in seven out of eleven human myeloid leukemia cell lines. Furthermore, in agreement with reports in esophageal SCC, treatment with 5-Aza, a specific inhibitor of DNA methylation, led to a significant increase in MT3 expression in AML cells (39.8 fold in HL-60 and 26.8 fold in MV4-11; *P* < 0.05 and *P* < 0.01, respectively). These results demonstrated that the MT3 promoter was consistently significantly methylated in human myeloid leukemia cells, suggesting that the MT3 promoter may also be methylated in pediatric AML.

Consistent with the cell line results, we found that the MT3 promoter was methylated in samples obtained from patients with pediatric AML. Aberrant methylation of MT3 was observed 39.0% (16/41) of pediatric AML samples compared to 6.7% (2/30) of NBM control samples. In addition, MT3 was significantly downregulated in the pediatric AML samples compared to the control samples. Further analysis revealed that patients with pediatric AML exhibiting methylated MT3 showed lower levels of MT3 expression compared to those with unmethylated MT3. These results confirmed that hypermethylation of the MT3 promoter occurs with high frequency in both AML cell lines and pediatric AML samples. However, comparisons between the two groups of patients showed no significant differences in MT3 methylation status and patient characteristics, including sex, age, initial hemoglobin level, white blood cell counts, platelet counts, and chromosomal abnormalities. Together, these results implied that various mechanisms may be involved in the downregulation of MT3 in pediatric AML, such as different post-transcriptional modifications, gene deletions, copy number reductions and histone code modifications. Further research will be required to elucidate the details of the underlying mechanisms.

A previous study in prostate cancer PC-3 cells revealed that overexpression of MT3 significantly increased cell proliferation, invasion and tumorigenic activities both *in vitro* and *in vivo*[31]. However, there were no reports on the role of MT3 in leukemia cells. In this study, we demonstrated that HL-60 and MV4-11 leukemia cells overexpressing MT3 inhibited cell proliferation in a dose-dependent manner. Further analysis by Annexin V assay revealed that there were a greater proportion of apoptotic cells in MT3-overexpressing leukemia cells compared to mock transfected cells. The apoptotic effect of MT3 in leukemia cells was confirmed by Western blot analysis which showed that MT3 overexpression led to enhanced expression of cleaved PARP, a marker of apoptosis. These findings suggested that MT3 may possess promising antitumor activity in AML cells.

Real-time PCR array analysis is an effective technique for quantifying the expression of a focused panel of genes [32,33]. Therefore, in order to explore the underlying mechanisms of MT3 antitumor activity, we carried out a real-time PCR array assay on 370 apoptosis-related genes in order to identify those genes that were dysregulated in AML following MT3 overexpression. The findings showed that 17 of the genes were significantly upregulated and 17 genes were significantly downregulated in MT3-overexpressing cells relative to control cells. The gene that was most significantly upregulated was found to be *FOXO1*, which belongs to the family of forkhead box transcription factors. These are downstream targets in the serine/threonine protein kinase B (PKB)/Akt pathway which is involved in the regulation of cell proliferation and survival [34-36]. It has been shown that withdrawal of growth factors leads to inactivation of the PI3K-Akt pathway, FOXO1 dephosphorylation at its Akt sites, nuclear translocation and activation of FOXO target genes [35-37]. FOXO1 plays a central role in initiating apoptosis by inducing expression of death genes, such as *FASL*. In the nucleus, FOXO1 is a key mediator of tumor suppression downstream of *PTEN*. Exogenous expression of *PTEN* induces FOXO1 to relocate to the nucleus, restoring its transcriptional activation. In addition, a constitutively active form of FOXO1 that cannot be phosphorylated by Akt has been found to induce apoptosis in *PTEN*-null cells, indicating that it has the same effect as reconstitution of *PTEN*[38]. Our results indicated that FOXO1 is a downstream target of MT3 in pediatric AML. A study in prostate cancer cells suggested that overexpression of FOXO1 resulted in apoptosis via increased expression of TRAIL (TNF-Related Apoptosis-Inducing Ligand) [39]. However, the mechanism and the role of these genes in MT3-induced apoptosis in AML remain to be elucidated.

## Conclusions

In this study, we identified epigenetic inactivation of MT3 in both AML cell lines and pediatric AML samples *via* hypermethylation of the MT3 promoter. Our findings also showed that transcriptional overexpression of MT3 could inhibit proliferation and induce apoptosis in AML cells. We identified 34 dysregulated apoptosis-related genes in MT3-overexpressing, including *FOXO1*. These results may provide new insights into the molecular mechanism of MT3-induced apoptosis; however, further research will be required to determine the underlying details. Together, our findings suggest that *MT3* may act as a putative tumor suppressor gene in pediatric AML.

## Competing interests

The authors declare that they have no competing interests.

## Authors’ contributions

PJ designed and directed the study. TYF and XLX finished the most of experiments. LZH and WNN finished the real-time PCR array. LYP, XYY, ZWL and CL collected the leukemia sample. XPF, HSY and LJ collected the clinical information of samples. DXJ and SLC supported the design of primer for BGS and MSP analysis. FF, LG and LYH drafted this manuscript. WJ, FX and NJ participated in study design and coordination, data analysis and interpretation and drafted the manuscript. All authors read and approved the final manuscript.

## Supplementary Material

Additional file 1 Analysis of promoter methylation in pediatric AML using NimbleGen Human DNA Methylation 385K Promoter Plus CpG Island Arrays. Click here for file
