# Supplementary figures and images for: Metallothionein III (MT3) is a putative tumor suppressor gene that is frequently inactivated in pediatric acute myeloid leukemia by promoter hypermethylation
Source: J Transl Med. 2014 Jun 25;12:182. doi: 10.1186/1479-5876-12-182 (PMC4082423; doi:10.1186/1479-5876-12-182)

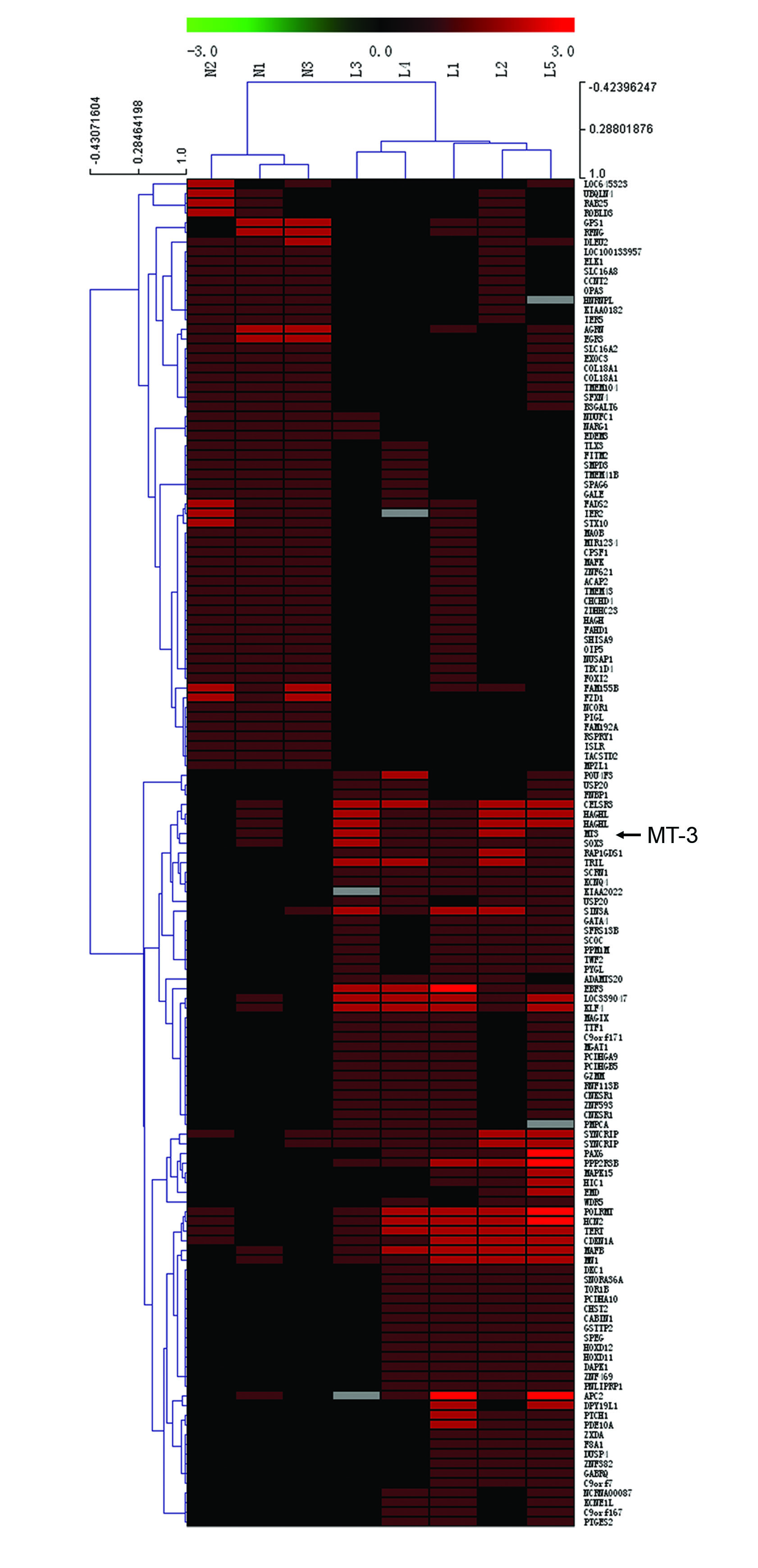

Supplement: Additional file 1 — Analysis of promoter methylation in pediatric AML using NimbleGen Human DNA Methylation 385K Promoter Plus CpG Island Arrays. [file 1479-5876-12-182-S1.jpeg]
